# EXPRESSION OF CONCERN: Cell Proliferation and Cell Cycle Alterations in Oesophageal p53‐Mutated Cancer Cells Treated With Cisplatin in Combination With Photodynamic Therapy

**DOI:** 10.1111/cpr.70241

**Published:** 2026-06-04

**EXPRESSION OF CONCERN**: C. Compagnin , M. Mognato , L. Celotti , G. Canti , G. Palumbo and E. Reddi , “Cell Proliferation and Cell Cycle Alterations in Oesophageal p53‐Mutated Cancer Cells Treated With Cisplatin in Combination With Photodynamic Therapy,” Cell Proliferation 43, no. 3 (2010): 262–274, 10.1111/j.1365-2184.2010.00673.x.20546244 PMC6496734

This Expression of Concern is for the above article, published online on 28 April 2010 in Wiley Online Library (wileyonlinelibrary.com), and has been issued by agreement between the journal Editor‐in‐Chief, Qi Zhou and John Wiley & Sons Ltd. The Expression of Concern has been agreed following concerns raised by a third party. An investigation identified duplications between the PDT/cyt c/pellet band and the CDDP + PDT/cyt c/pellet band presented in Figure 9. A further duplication was observed between the CTR/BCL‐2/pellet band and the CDDP + PDT/BCL‐2/pellet band, also in Figure 9. The authors were contacted for comment and asked to provide supporting data. Due to the time that has elapsed since publication, the original data were no longer available. Although the concerns cannot be fully resolved, the journal considers that the overall conclusions of the article are not affected. The journal has therefore decided to issue an Expression of Concern to inform and alert readers.

